# Associations of tumor regression grade with outcomes in patients with locally advanced rectal cancer treated with preoperative two-week course of radiotherapy

**DOI:** 10.18632/oncotarget.22118

**Published:** 2017-10-26

**Authors:** Yong-Heng Li, Jin-Luan Li, Xiang-Gao Zhu, Jun-Yan He, Li-Mei Lin, Xiao-Yi Lin, Li-Rui Tang, Yong Cai

**Affiliations:** ^1^ Department of Radiation Oncology, Key Laboratory of Carcinogenesis and Translational Research (Ministry of Education /Beijing), Peking University Cancer Hospital & Institute, Beijing, China; ^2^ Departments of Radiation Oncology, Fujian Medical University Cancer Hospital, Fujian Cancer Hospital, Fuzhou, China; ^3^ Department of Biochemistry and Molecular Biology, University of South China, Hengyang, China; ^4^ Affiliated Xiamen First Hospital of Xiamen University, Xiamen, China; ^5^ Xiamen Humanity Hospital, Xiamen, China

**Keywords:** tumor regression grade, two-week course, radiotherapy, rectal cancer, prognosis

## Abstract

**Purpose:**

Studies concerning tumor regression grade (TRG) after two-week course of radiotherapy (RT) are limited. We tried to assess associations of TRG and outcomes in patients with locally advanced rectal cancer (LARC) treated with preoperative two-week course of RT.

**Methods:**

356 consecutive LARC patients were retrospectively assessed. Patients with complete/intermediate (TRG1-3) and poor (TRG4-5) regressions were compared for overall survival (OS), disease-free survival (DFS) and metastasis-free survival (MFS).

**Results:**

By univariate analysis, pretreatment and postoperative factors including TNM stages, ypT, ypN, surgical procedure, pathological grade, and TRG impacted survival outcomes. Complete/intermediate regressions (TRG1-3) had significantly improved survival outcomes compared with poor ones (TRG4-5) (5y-OS, 85.8% vs. 65.8%, P=0.001; 5y-DFS, 76.0% vs. 53.7%, P<0.001; 5y-MFS, 84.2% vs. 66.7%, P<0.001). Multivariate analysis showed that ypN (P<0.001) and pathological grade (P=0.018) were the most important independent prognostic factors for DFS. ypT (P=0.014) and ypN (P=0.001) were the independent prognostic factors for MFS. Meanwhile, ypT (P=0.009), ypN (P=0.001), surgical procedure (p=0.001), and TRG (p=0.019) were the independent prognostic factors for OS.

**Conclusions:**

Complete/intermediate TRG regressions had a more favorable prognosis than the poor group. When treated with preoperative two-week course of RT; ypT, ypN, surgical procedure, and TRG seem to affect OS.

## INTRODUCTION

Rectal cancer is the third most common malignancy worldwide [1]. Pre-operative radiotherapy (pre-RT) followed by total mesorectal excision (TME) has become the standard treatment sequence for locally advanced rectal cancer (LARC) [2, 3]. Clinical trials have largely revealed the best regimens for pre-RT. In China, a modified 30Gy/10 fraction protocol for pre-RT was recommended by the Chinese Anti-Cancer Association in 2001 [4]. The modified protocol offers similar clinical outcomes to the reported long course RT regimen, providing an alternative to pre-RT regimens in China [5].

Tumor regression grade (TRG) after pre-RT was found to be significantly correlated with long term outcome in several studies [6-9]. However, TRG varies from complete absence of tumor cells to little or no regressive changes. Further studies suggested that complete response after pre-RT might improve survival [10-12]. Moreover, Suarez et al. [13] concluded that TRG might be a better prognostic factor than downstaging in predicting disease-free survival after pre-chemoradiotherapy (CRT). Indeed, evaluation of TRG has been recommended as a routine procedure for rectal cancer [14].

While multiple reports have assessed the prognostic value of TRG in rectal cancer after long- or short-term pre-RT, studies evaluating TRG after 30-Gy protocol pre-RT are scarce. This study aimed to evaluate the association of TRG with long-term outcomes in patients with rectal cancer treated with 30 Gy/10 fraction pre-RT.

## RESULTS

### Patient characteristics

A total of 356 patients with mid-low (within 10 cm to the anal verge) rectal adenocarcinoma treated with pre-RT were assessed. The patient characteristics are summarized in Table 1. Median patient age was 58 years (range, 22 to 80 years). There were 205 (57.6%) male and 151 (42.4%) female patients. Among them, 216 (60.7%) patients had low rectal cancer (≤ within 5 cm to the anal verge) and 140 (39.3%) presented with mid-rectal cancer (5 -10 cm). Median distance to the anal verge was 5 cm (range, 1 to 10 cm). Median time interval to surgery was 18 days, consisted of 162 (45.5%) longer time inteval than 18 days and 194 (54.5%) shorter than 18 days. Clinical stages before radiotherapy included stage I (n=8, 2.2%), stage II (n=51, 14.3%), stage III (n=286, 80.3%) and uncertain preoperative staging (n=11). Of the 356 patients, 262 (70.2%) received postoperative adjuvant chemotherapy.

**Table 1:** Patient characteristics

Characteristic	N (%)
Age (years)	
≤60	205 (57.6)
>60	151 (42.4)
Sex	
Male	150 (41.6)
Female	206 (58.4)
Distance to anal verge (cm)	
0 - 5	216 (60.7)
>5 -10	140 (39.3)
Pre-operate staging	
I	8 (2.2)
II	51 (14.3)
III	286 (80.3)
Surgery method	
LAR	234 (65.7)
APR + other	122 (34.3)
Time interval to surgery	
≤Median (18 days)	162(45.5)
>Median	194(54.5)
Tumor regression grade	
1	17 (4.8)
2	142 (39.9)
3	22 (6.2)
4	13 (3.6)
5	162 (45.5)

*APR* abdominoperineal resection; *LAR* low anterior resection.

### Disease progression

Median follow-up for all patients was 66.5 months (range, 5.1 to 131.7 months). Of the 356 analyzed patients, there were 86 (24.2%) deaths, 38 (10.7%) locoregional recurrences, and 77 (21.6%) distant metastases. The rate of 5-year locoregional recurrences and distant metastases were 5.3% and 10.7%. Locoregional recurrence cases included 19 (5.3%, 19/356) patients with pelvic recurrence; 11 (3.1%, 11/356) and 8 (2.2%, 8/356) patients had pre-sacral and anastomotic recurrence, respectively. Common distant metastasis sites included lung (7.0%, 25/356), liver (6.5%, 23/356) and bone (2.5%, 9/356).

### TRG and association with clinicopathologic factors

Tumor regression grades were TRG 1 (n=17, 4.8%), TRG 2 (n=142, 39.9%), TRG 3 (n=22, 6.2%), TRG 4 (n=13, 3.6%), and TRG 5 (n=162, 45.5%). To simplify the analysis, TRGs were combined into two groups, including complete/intermediate (TRG 1-3) and poor (TRG 4-5) regression groups. The associations of TRG with clinicopathologic factors are summarized in Table 2. TRG was significantly associated with age, clinical T stage, clinical N stage, ypT stage, ypN stage, American Joint Committee on Cancer (AJCC) cancer staging seventh edition, [15] pathologic grade, and lymphatic invasion. There were no significant differences between the two groups in patient sex and time interval to surgery. The relationship between TRG and pN was analyzed. Overall, 180 patients (50.6%) with TRG1-3 were pN0, and one (0.3%) was pN+. Meanwhile, 17 patients (4.8%) with TRG 4-5 were pN0 and the remaining 158 (44.4%) were pN+ (P<0.001). Therefore, 99.4% (180/181) of TRG 1-3 patients achieved pN0, for only 9.7% (17/175) of TRG 4-5 cases.

**Table 2:** Association of TRG with clinicopathologic factors in 356 patients

Characteristic	TRG 1-3	TRG 4-5	P
	No. (%)	No. (%)	
Age (years)			0.002
≤60	90 (25.3)	116 (32.6)	
>60	91 (25.6)	59 (16.6)	
Sex			0.306
Male	109 (30.6)	96 (27.0)	
Female	72 (20.2)	79 (22.2)	
cT			0.003
cT1-2	19 (5.3)	5 (1.4)	
cT3-4	154 (43.3)	167 (46.9)	
Unknown	8 (2.2)	3 (3.1)	
cN			0.001
N0	41 (11.5)	18 (5.1)	
N1/2	132 (37.1)	154 (43.3)	
Unknown	8 (2.2)	3 (0.8)	
ypT category			<0.001
T0	17 (4.8)	2 (0.6)	
T1	17 (4.8)	3 (0.8)	
T2	71 (19.9)	26 (7.3)	
T3	75 (21.1)	140 (39.3)	
T4	1 (0.3)	4 (1.1)	
ypN category			<0.001
N0	180 (50.6)	17 (4.8)	
N+	1 (0.3)	158 (44.4)	
AJCC TNM stage			<0.001
pCR	17 (4.8)	0 (0)	
I	88 (24.7)	4 (1.1)	
II	75 (21.1)	13 (3.7)	
III	1 (0.3)	158 (44.7)	
Pathologic grade			<0.001
High	3 (0.8)	1 (0.3)	
Moderate	132 (37.1)	108 (30.3)	
Low	28 (7.9)	63 (17.7)	
Others	18(5.1)	3(0.8)	
Time interval to surgery			0.0651
≤Median (18day)	121 (34)	113 (31.7)	
>Median	60 (16.9)	62 (35.4)	
Lymphatic invasion			<0.001
L0	170 (47.8)	142 (39.9)	
L1	11 (3.1)	33 (9.3)	

*TRG* tumor regression grade.

### TRG is a prognostic factor for OS, DFS and MFS

**Figure 1:**
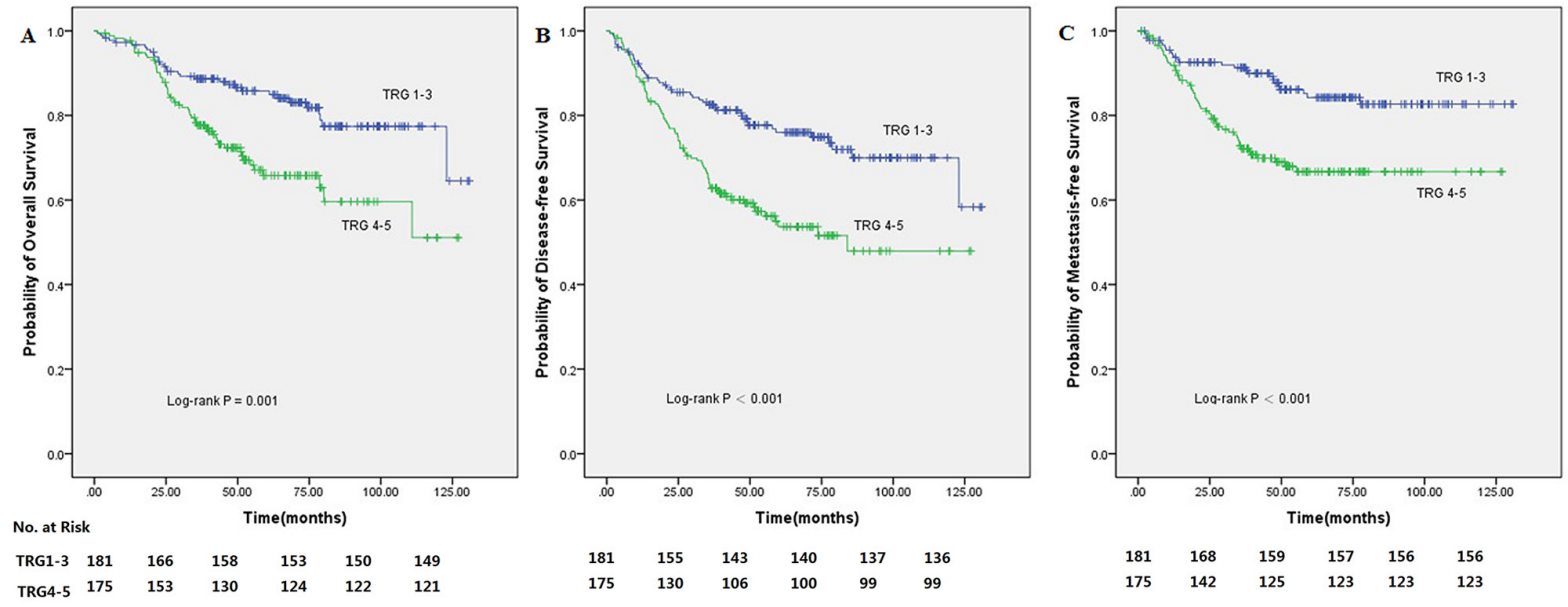
Kaplan-Meier analysis of 356 rectal cancer patients treated with preoperative radiotherapy (30 Gy in 10 fractions) followed by surgery with curative intent according to tumor regression grade (TRG1-3/TRG4-5) showed statistically significant difference **(A)** The 5-year OS of patients with TRG1-3 versus TRG4-5. **(B)** The 5-year DFS of patients with TRG1-3 versus TRG4-5. **(C)** The 5-year MFS of patients with TRG1-3 versus TRG4-5.

**Figure 2:**
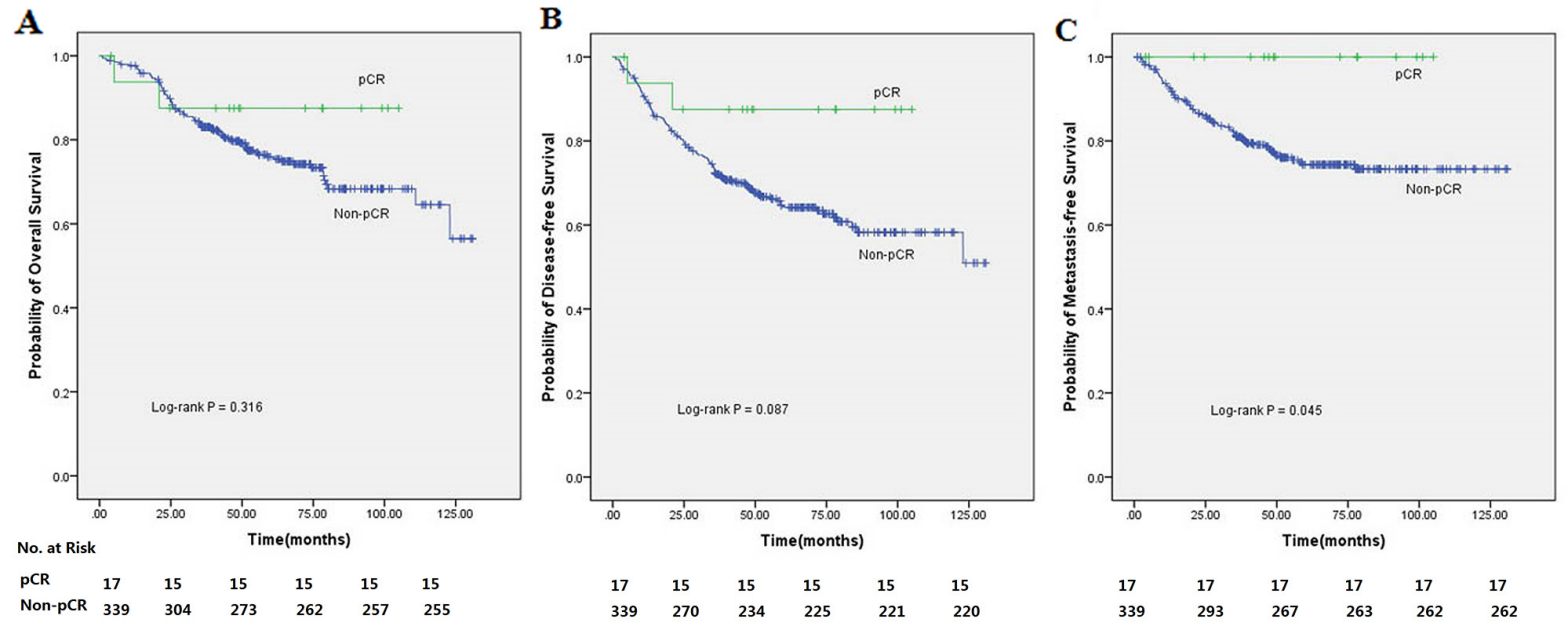
Kaplan-Meier analysis of 356 rectal cancer patients treated with preoperative radiotherapy (30 Gy in 10 fractions) followed by surgery with curative intent according to pCR (pCR/non-pCR) **(A)** The 5-year OS of patients with pCR versus non-pCR. **(B)** The 5-year DFS of patients with pCR versus non-pCR. **(C)** The 5-year MFS of patients with pCR versus non-pCR.

The TRG 1-3 and TRG 4-5 groups showed statistically significant differences in patient outcomes (Figure 1). Five-year OS rates were 85.8% (95%CI=80.5-91.1%) and 65.8% (95%CI=57.8-73.8%), respectively (P=0.001). Five-year DFS rates were 76.0% and 53.7% (P<0.001), and five-year MFS rates were 84.2% and 66.7% (P<0.001), respectively. In addition, patients in the pathologic complete remission (pCR) and non-pCR groups showed five-year OS rates of 87.5% and 75.9%, and five-year DFS rates of 87.5% and 64.2%, respectively. The pCR group showed significantly improved five-year MFS compared with the non-pCR group (100% vs. 74.4%, P=0.045) (Figure 2). Although not reaching statistical significance, the pCR group also showed a trend toward improved OS and DFS rates compared with the non-pCR group.

By univariate analysis, TRG was found to be significantly correlated with OS, DFS and MFS (Figure 1). As shown in Table 3, other factors significantly associated with DFS by univariate analysis included the ypT and ypN categories (P<0.001), TNM stage (P<0.001), and pathologic grade (P=0.002). Besides TRG, other factors in Table 4 that were significantly correlated with OS included the ypT and ypN categories (P<0.001), TNM stage (P<0.001), pathologic grade (P=0.006), and the surgical method (P=0.007). Additionally, TRG (P<0.001), the ypT and ypN categories (P<0.001), TNM stage (P<0.001), and pathologic grade (P<0.001) also significantly affected MFS by univariate analysis (Table 5).

**Table 3:** Cox regression for disease-free survival analysis

	Univariate			Multivariate		
	HR	95%CI	P	HR	95%CI	P
Age	0.745	0.498-1.116	0.154			
Sex	0.871	0.590-1.286	0.487			
cT	2.100	0.772-5.711	0.146			
cN	1.440	0.804-2.581	0.220			
Distance to anal verge (cm)	1.137	0.766-1.686	0.525			
ypT	2.731	1.701-4.385	<0.001	1.636	0.879-3.047	0.121
ypN	2.671	1.780-4.009	<0.001	2.285	1.512-3.452	<0.001
AJCC TNM stage	1.862	1.447-2.397	<0.001	1.076	0.545-2.125	0.833
TRG 1-3 vs. TRG 4-5	2.259	1.501-3.399	<0.001	0.446	0.139-1.434	0.175
Pathologic grade	1.889	1.275-2.798	0.002	1.620	1.087-2.415	0.018
Surgery method	1.228	0.818-1.844	0.322			
Time interval to surgery	1.428	0.968-2.106	0.073			
Lymphatic invasion	1.337	0.784-2.280	0.287			
Adjuvant Chemotherapy	1.410	0.898-2.216	0.136			

*TRG* tumor regression grade.

**Table 4:** Cox regression for overall survival analysis

	Univariate			Multivariate		
	HR	95%CI	P	HR	95%CI	P
Age	0.957	0.623-1.469	0.841			
Sex	1.118	0.727-1.720	0.612			
cT	2.366	0.747-7.496	0.143			
cN	1.508	0.798-2.853	0.206			
Distance to anal verge (cm)	0.764	0.488-1.197	0.240			
ypT	2.503	1.512-4.144	<0.001	2.141	1.214-3.777	0.009
ypN	2.721	1.746-4.240	<0.001	7.774	2.360-25.601	0.001
AJCC TNM stage	1.825	1.390-2.397	<0.001	1.086	0.482-2.444	0.843
TRG 1-3 vs. TRG 4-5	2.143	1.378-3.333	0.001	1.647	1.076-2.492	0.019
Pathologic grade	1.849	1.192-2.867	0.006	1.221	0.833-1.788	0.307
Surgery method	0.744	0.601-0.922	0.007	0.483	0.310-0.753	0.001
Time interval to surgery	1.443	0.942-2.210	0.092			
Lymphatic invasion	1.482	0.848-2.591	0.167			
Adjuvant Chemotherapy	0.709	0.451-1.114	0.136			

*TRG* tumor regression grade.

**Table 5:** Cox regression for metastasis-free survival analysis

	Univariate			Multivariate		
	HR	95%CI	P	HR	95%CI	P
Age	0.714	0.447-1.141	0.159			
Sex	1.321	0.845-2.065	0.222			
cT	2.084	0.657-6.616	0.213			
cN	1.406	0.722-2.736	0.316			
Distance to anal verge (cm)	1.028	0.906-1.166	0.673			
ypT	2.954	1.679-5.197	<0.001	2.120	1.167-3.851	0.014
ypN	3.060	1.896-4.939	<0.001	2.406	1.451-3.990	0.001
AJCC TNM stage	1.985	1.467-2.686	<0.001	0.862	0.355-2.091	0.084
TRG 1-3 vs. TRG 4-5	2.458	1.523-3.966	<0.001	0.336	0.085-1.326	0.129
Pathologic grade	1.108	0.781-1.573	0.564			
Surgery method	0.999	0.617-1.619	0.997			
Time interval to surgery	1.234	0.789-1.930	0.357			
Lymphatic invasion	1.601	0.897-2.857	0.111			
Adjuvant Chemotherapy	0.854	0.517-1.412	0.539			

*TRG* tumor regression grade.

All significant factors in univariate analysis were entered in multivariate analysis for the respective end points. We found that ypN (HR=2.285, 95%CI=1.512-3.452, P<0.001) and pathological grade (HR=1.620, 95%CI=1.087-2.415, P=0.018) were the most important independent prognostic factors for DFS. ypT (HR=2.120, 95%CI=1.167-3.851, P=0.014) and ypN (HR=2.406, 95%CI=1.451-3.990, P=0.001) were the independent prognostic factors for MFS. Meanwhile, ypT (HR=2.141, 95%CI=1.214-3.777, P=0.009), ypN (HR=7.774, 95%CI=2.360-25.601, P=0.001), surgical procedure (HR=0.483, 95%CI=0.310-0.753, P=0.001), and TRG (HR=1.647, 95%CI=1.076-2.492, P=0.019) were the independent prognostic factors for OS. AS seen in Table 3-5.

## DISCUSSION

Here, we report the largest cohort study assessing the associations of TRG with long-term outcomes in LARC patients treated with preoperative two-week course of radiotherapy. The results showed that complete/intermediate TRG responders had a favorable prognosis. In addition, ypT, ypN, surgical procedure, and TRG seemed to affect OS.

The association of TRG after pre-RT with prognosis has been widely discussed for long-term or short-term RT regimens [16]. However, studies evaluating TRG in preoperative two-week course of radiotherapy are limited. We firstly reported the efficacy of a two-week course of pre-RT with 30 Gy in 10 fractions, and its associated clinical prognostic factors affecting OS and DFS in LARC [5]. Interestingly, patients treated with this modified regimen were shown to achieve similar OS to the reported long course RT regimen. After adding TRG to the long-term analysis, we further found that TRG might affect OS.

Besides the Mandard scoring system, several tumor regression systems have been recommended [17-20]. The choice of tumor regression system remains controversial. Lossi et al. [20] used the Dworak system to evaluate the correlation between TRG and DFS, and found that good TRG could predict better DFS in LARC cases treated with long-course pre-CRT. Dworak et al [19] applied a 5-point scoring system of 0 to 4, ranging from no regression (TRG 0) to total regression (TRG 4), which is similar to Mandard’s. Wheeler et al. [17] proposed another rectal cancer regression grade (RCRG) and modified Mandard classification into 3 points: RCRG 1, either pCR or only microscopic foci of adenocarcinoma remaining; RCRG 2, marked fibrosis but macroscopic disease present; RCRG 3, poor response with little or no fibrosis, and abundant macroscopic disease. Rodel et al. [18] also suggested primary tumor regression to be grouped into three categories, from complete regression (Grade 1) to poor regression (Grade 3). In this study, application of Mandard system successfully identified two subgroups with different prognoses (5y-OS, 85.8% vs.65.8%, P=0.001). Furthermore, TRG was one of the most important independent prognostic factors for OS.

The association of TRG with DFS has been demonstrated in previous reports. Losi et al. [20] demonstrated that TRG (Dworak grade) does not have a prognostic value for DFS in patients with residual cancer. Beddy et al. [21] applied a simplified Mandard system (3-point TRG) and found improved DFS in the combined group of patients with either complete- or near complete response versus the remaining patients. However, no difference in DFS was found between the TRG2 and TRG3 groups. Similarly, Bouzouirene et al. [6] and Rodel et al. [18] found better DFS with good tumor responders, as reflected by TRG categories in univariable analyses. However, after adjusting for other confounding variables, TRG showed no independent impact on DFS in multivariate analysis. In contrast, Vecchio et al. [16] and Dhadda et al. [22] both observed an association of TRG with DFS in multivariable analyses. In addition, other reports with different TRG systems and multivariate analyses failed to demonstrate the prognostic value of TRG for DFS [18, 23-25]. In the current analysis, however, five TRG categories were combined into two different groups, including complete/intermediate (TRG 1-3) and poor (TRG 4-5) responders. The responder groups showed significantly different DFS rates (5y-DFS, 76.0% vs.53.7%, P<0.001).

The positive association between TRG and the risk of nodal disease was described by univariable analysis in this study and others [6, 16, 18, 21, 26]. As shown above, complete/intermediate tumor regression (TRG 1-3) was associated with improved disease control in lymph nodes (ypN positive, 0.3%), which might account for DFS. Patients with poor tumor regression (TRG 4-5) had a higher risk of lymph node involvement (ypN positive, 44.4%) and an unfavorable outcome. In addition, histopathologic factors, especially the N stage, remained the most important prognostic factors in the multivariate model, corroborating previous studies [18, 27]. After pre-CRT, positive lymph nodes could both indicate primary cancer aggressiveness and resistance to CRT. Therefore, patients with ypN positive would have an unfavorable prognosis irrespective of the TRG.

This study demonstrated that tumor regression after pre-RT was closely associated with pathological T and N stages. As shown above, the majority of poorly responding tumors contributed to nodal metastasis, indicating that TRG could be an effective supplement to the TNM classification. As a prognostic factor after pre-RT, TRG might also guide clinical decision making for further postoperative adjuvant chemotherapy in different subgroups of patients. Further prospective clinical trials focusing on the predictive value of TRG for adjuvant chemotherapy are warranted.

As a single-center study, some limitations should be mentioned. First, this was a retrospective study, which might have selection bias. Sample size in some TRG subgroups was relatively small; this might affect the reliability of the findings. In addition, TRG requires surgery and can only be used after pathology. Several studies had investigated the value of other indicators such as dynamic contrast enhanced magnetic resonance imaging (DCE-MRI) or blood count levels for treatment response assessment [28-31]. Therefore, further studies are necessary to explore tumor response to preoperative two-week course of radiotherapy by combination of TRG and other indicators.

## MATERIALS AND METHODS

### Patients

A total of 356 LARC patients who underwent a 30-Gy pre-RT followed by curative surgery in our institution from September 2002 to October 2010 were analyzed in this study. Eligible patients were selected according to the following criteria: (1) pathological diagnosis of adenocarcinoma; (2) middle or low rectum (within 10 cm of the anal verge) involvement, (3) locally advanced disease (clinically T3/T4, or any T category and N positive) revealed by endorectal ultrasound (EUS) and MRI, a few patients presenting with T1/T2N0 tumors located within 5 cm from the anal verge could also be included for the purpose of sphincter-preserving. (4) no history of other malignant diseases, and (5) no distant metastasis. Exclusion criteria were: (1) familial adenomatous polyposis; (2) upper rectal cancer; (3) a history of other malignancies within 5 years. Pretreatment evaluation included a complete history, physical examination, complete laboratory tests, and preoperative staging. The study was approved by the Ethics Committee of Beijing Cancer Hospital, Beijing, China. And all the eligible patients signed the informed consent before treatment.

## TREATMENT

The RT was delivered in 10 fractions of 3 Gy, five times per week over 2 weeks. This dose was delivered using a 3-field technique, with the patient in the prone position. The clinical target volume included the primary tumor, anorectum, and mesorectal, perirectal, and internal iliac nodes, but excluding the external and common iliac nodes. The delineations of the 3 pelvic fields were described previously [32, 33].

All the patients underwent total mesorectal excision (TME) 2 weeks after pre-RT completion. The choice between abdominoperineal resection and anterior resection was left to the discretion of the attending surgeon. The decision for adjuvant chemotherapy was left to medical oncologists.

### Tumor regression grade

**Figure 3:**
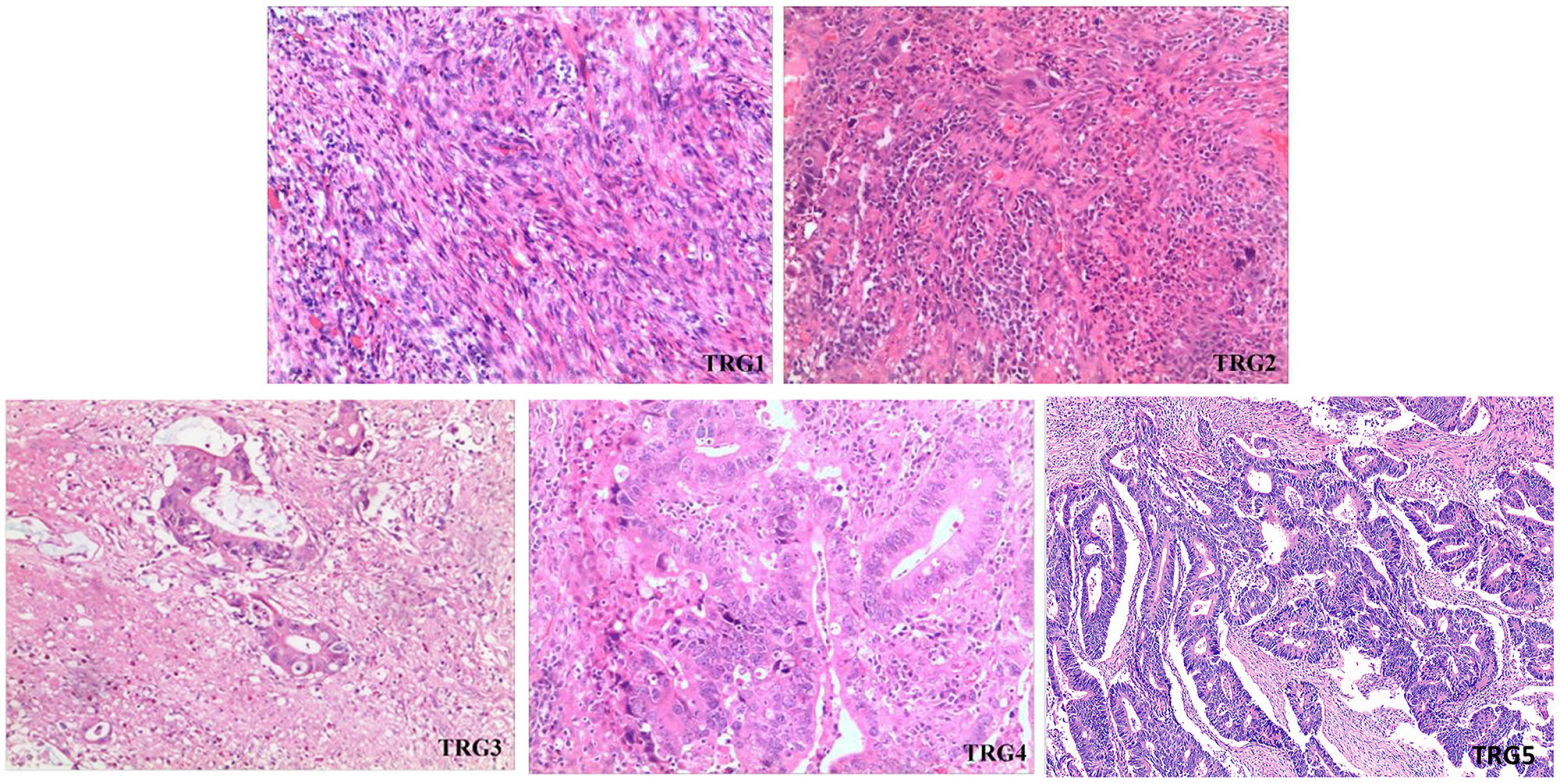
Tumor regression grading of rectal tumors in patients treated preoperatively with radiotherapy (TRG1) complete response with no residual cancer or fibrosis extending through the wall; (TRG2) presence of residual cancer cells scattered through the fibrosis; (TRG3) increased number of residual cancer cells, with fibrosis predominating; (TRG4) residual cancer outgrowing the fibrosis; (TRG5) absence of regressive changes.

All resection specimens were assessed independently by two pathologists, who were aware of the patient’s history but blinded to clinical stage. The histology of all surgical specimens was classified according to the Mandard TRG system [34]: TRG1, complete response with no residual cancer or fibrosis extending through the wall; TRG2, presence of residual cancer cells scattered through the fibrosis; TRG3, increased number of residual cancer cells, with fibrosis predominating; TRG4, residual cancer outgrowing the fibrosis; TRG5, absence of regressive changes. As shown in Figure 3.

### Follow-up

Patients were routinely followed up every 3 months for the first 2 years, every 6 months for subsequent 3 years, and annually thereafter. Follow-up laboratory tests included complete blood count, blood chemical analysis, and carcinoembryonic antigen (CEA) detection. Chest x-ray, abdominal ultrasound or CT, and CT of the pelvis were performed at each follow-up visit.

### Statistical analysis

The primary endpoints of this study included overall survival (OS), disease-free survival (DFS) and metastasis-free survival (MFS). Categorical variables were compared by Pearson χ^2^ test. Survival curves were generated by the Kaplan-Meier method, and compared using the log-rank test. Two-tailed P<0.05 was considered statistically significant. The Cox proportional hazards model was used for multivariate analysis. Statistical analyses were performed with SPSS version 22.0.

## CONCLUSIONS

In summary, TRG appears to be a good prognostic factor for patients treated with preoperative two-week course of radiotherapy. More prospective trials are required to validate these findings and assess TRG for its promising value in clinical decision making for adjuvant therapy.

### Ethical Approval

The study was approved by the Ethics Committee of Beijing Cancer Hospital, Beijing, China. All procedures performed in studies involving human participants were in accordance with the ethical standards of the institutional and/or national research committee and with the 1964 Helsinki declaration and its later amendments or comparable ethical standards.
